# Mastering your fellowship: Part 3, 2024

**DOI:** 10.4102/safp.v66i1.5886

**Published:** 2024-02-29

**Authors:** Klaus B. von Pressentin, Ts’epo Motsohi, Gert Marincowitz, Tasleem Ras

**Affiliations:** 1 Division of Family Medicine, Department of Family, Community and Emergency Care, Faculty of Health Sciences, University of Cape Town, Cape Town, South Africa; 2 Department of Family and Emergency Medicine, Stellenbosch University, Cape Town, South Africa; 3 Department of Family Medicine, University of Limpopo, Polokwane, South Africa; 4 Department of Family, Community and Emergency Care, Faculty of Health Sciences, University of Cape Town, Cape Town, South Africa

**Keywords:** family physicians, FCFP (SA) examination, family medicine registrars, postgraduate training, national exit examination, general adult medicine

## Abstract

The series ‘Mastering your Fellowship’ provides examples of the question formats encountered in the written and clinical examinations, Part A of the Fellowship of the College of Family Physicians of South Africa (FCFP [SA]) examination. The series aims to help family medicine registrars (and supervisors) prepare for this examination.

This *South African Family Practice* journal section aims to help registrars prepare for the Fellowship of the College of Family Physicians of South Africa (FCFP [SA]) Final Part A examination. It will provide examples of the question formats encountered in the written exam: multiple choice question (MCQ) in the form of single best answer (SBA – Type A) and extended matching question (EMQ – Type R); short answer question (SAQ), questions based on the critical reading of a journal article (CRJ: evidence-based medicine) and an example of an objectively structured clinical examination (OSCE) question. Each of these question types is presented based on the College of Family Physicians blueprint and the key learning outcomes of the FCFP (SA) programme. The MCQs draw on the 10 clinical domains of family medicine, the SAQs align with the five national unit standards, and the critical reading section includes evidence-based medicine and primary care research methods.

This edition aligns with unit standard two (Evaluate and manage patients with both undifferentiated and more specific problems cost-effectively according to the biopsychosocial approach), unit standard three (Improve the health and quality of life of the community), unit standard four (Facilitate the learning of others regarding the discipline of family medicine, primary healthcare and other health-related matters) and unit standard five (Conduct all aspects of healthcare in an ethical and professional manner). The clinical domain covered in this edition is general adult medicine. We suggest you attempt to answer the questions (by yourself or with peers or supervisors) before finding the model answers online: http://www.safpj.co.za/.

Please visit the Colleges of Medicine website for guidelines on the Fellowship examination: https://www.cmsa.co.za/view_exam.aspx?QualificationID=9.

We are keen to hear about how this series assists registrars and their supervisors prepare for the FCFP (SA) examination. Please email us (editor@safpj.co.za) with your feedback and suggestions.

## Multiple choice question (MCQ)

A 51-year-old man presents to the community health centre with a 2-month history of marked weight loss, polyuria and polydipsia. On examination, his random serum glucose = 24 mmol/L, urine dipsticks = 4+ glucose, blood pressure = 126/30 mm/Hg, heart rate = 78 beats per minute, and his body mass index (BMI) is 21 kg/m^2^. The rest of his examination is normal. You decide to start him on metformin and simvastatin.

Which one of the following is the most appropriate additional medication to prescribe for him?

- Glimepiride
- Glibenclamide
- Long-acting insulin at night
- Biphasic insulin
- Basal Bolus Insulin

*Answer: d*)

## Discussion

The patient presents in a catabolic state, which can occur with type 2 diabetes. This is one of the metabolic decompensation criteria for which insulin is recommended as first-line treatment. The other criteria include the following:

- Random glucose levels consistently > 16.5 mmol/L
- Fasting plasma glucose levels > 14 mmol/L
- Haemoglobin A1C (HbA1C) > 10%
- Presence of persistent ketogenesis, ketoacidosis or hyperosmolar non-ketotic state

While the basal-bolus regimen typically given to patients with type 1 diabetes is also recommended with an equally high level of evidence, the basal-bolus regimen is arguably more an individualised treatment option for a patient with type 2 diabetes who is being initiated on insulin at the point of diagnosis. The choice of insulin regimen should be tailored to accommodate the patient’s daily activities, conditions and socioeconomic circumstances and should not be a one-size-fits-all approach. Early insulin initiation, optimisation and patient empowerment can help manage type 2 diabetes effectively but will require professional education and upskilling in the practical management of diabetes.

**Further reading**

- Chapter 10: Glucose control: Insulin therapy. In: SEMSDA Type 2 Diabetes Guidelines Expert Committee. The 2017 SEMDSA Guideline for the Management of Type 2 Diabetes. JEMDSA [serial online]. 2017 [cited 2023 Dec 05];22(Suppl. 1):S49–S50. Available from: https://www.semdsa.org.za/for-members/guidelines
- Coetzee A. An introduction to insulin use in type 2 diabetes mellitus. S Afr Fam Pract. 2023;65(2):a5702.

## Short answer question (SAQ): Community-orientated primary care in adult medicine

*This question was previously used in an FCFP(SA) written paper*.

The operational manager at a clinic where you are the family physician mentions that she is concerned about the increased number of patients they are diagnosing with diabetes mellitus lately at the clinic. The clinic provides community-oriented primary care. She asks you to assist her with strategies to address the problem.

1. Explain how the principles of COPC could be incorporated into your planning. (6 marks)
2. Mention and describe the role of any six categories of health workers you want to include in your local health team to work with you on the problem. (6 marks)
3. Explain how you will do the local health analysis to assess the extent of the problem in the area served by the clinic. (3 marks)
4. There is currently no community health forum in the area. Identify stakeholders and their roles to join the health team who should be involved in establishing the forum. Mention any six stakeholders. (6 marks)
5. You and the local health team identify the two main contributors to the problem: late presentation/delayed diagnosis of diabetes mellitus and the high prevalence of obesity in the community. Suggest two practical community-based activities to address each of the problems. (4 marks)

**Total: 25 marks**

*Suggested answers (the answers should show some application to the scenario)*

### 1. Explain how the principles of COPC could be incorporated into your planning

- **Local health analysis:** Evaluate how many diabetic patients are in the area (attending the clinic) and how well they are controlled. Evaluate the number of diabetic patients identified by the community health worker (CHW) during household registration.
- **Local institutional analysis:** Identify all institutions other than the clinic involved or potentially involved in the care of diabetic patients, such as private practices, health non-governmental organisation (NGO), traditional authorities, municipal representatives, churches, sports clubs and other government sectors.
- **Comprehensive care:** Services at the clinic should include care for diabetic patients with acute problems, follow-up, repeat prescriptions, health promotion at the clinic level, health promotion in the community, home-based diabetic care by CHW/ward-based outreach team (WBOT), early identification plans at the clinic and in the community.
- **Equity:** Acute care should not have preference over chronic care. Give particular attention to patients who miss appointments, those who are disabled, elderly and vulnerable patients such as child-headed households or impoverished patients.
- **Practise with science:** Diabetic care at the clinic should be based on the latest essential drug list (EDL) guidelines (and SEMDSA guidelines).
- **Service integration around users:** The health service/clinic should implement plans to involve the community in the clinic’s management. The most important would be an active clinic committee. Also, involving patients in committees and projects. (6)

### 2. Mention and describe the roles of any six categories of health workers you want to include in your local health team to work with you on the problem

- **Operational manager of the clinic:** Link with management and administration for access to resources.
- **Nurses** (professional nurse and nurse doing observations): Dealing with clinic patients and assessing their parameters.
- **CHWs and their team leader:** Front-line workers with patient contact at the household level.
- **Patients:** Give the team patient perspectives.
- **Dietician, nutritionist/nutrition advisors:** Assist with nutrition education.
- **Health educators/health promotion officers:** Assist with health education strategies.
- **Family physician/doctor/private GP/clinical associates:** As clinical experts, assist with patient management plans and train other team members.
- **Pharmacist:** Assist with drug supplies.
- **Clinic administrative personnel:** Assist with files and records.
- **Physiotherapist**: Assist with exercise programmes. (6)

### 3. Explain how you will do the local health analysis to assess the extent of the problem in the area served by the clinic

- **Identify the number of diabetic patients attending the clinic:** Check the attendance register, number of chronic scripts with diabetic medication, diabetic patient register (if your clinic has such) and clinic statistics. Ask CHW to check how many patients with DM are among the households they care for. Check how many diabetic patients they have on their household registrations. Do a household survey using CHW if they have not registered all households.
- **Compare the number of patients with the population** attending the clinic to calculate a ratio indicative of the prevalence of DM in the community.
- **Compare this ratio** (prevalence) with other areas to quantify the extent of the problem in the community served by the clinic. (3)

### 4. There is currently no community health forum in the area. Identify stakeholders and their roles in the health team who should be involved in establishing the forum

- **Traditional leaders:** Leaders in the community and opinion makers.
- **Municipality/ward counsellor/political leaders:** Leaders in the community and opinion makers access resources.
- **Political parties:** Leaders in the community shape opinions.
- **Traditional healers:** They could identify patients early and refer them to the clinic.
- **NGOs, especially health and sports NGOs:** They help with programmes to promote exercise and access resources.
- **Cultural/community organisations:** Participate in education and mobilise people.
- **Churches/religious leaders/faith-based organisations:** Leaders and opinion makers help with community education.
- **Private health practitioners:** They are also caretakers of patients.
- **Other government sectors:** Social workers, community development, agriculture, sports and recreation, police: assist with resources and support. (6)

### 5. You and the local health team identify the two main contributors to the problem: Late presentation/delayed diagnosis of diabetes mellitus and the high prevalence of obesity in the community

*Suggest two practical community-based activities to address each problem*:

- Late presentation: (2)
  - CHW door-to-door campaign with a diabetes screening tool and preferably a glucometer to screen people at home for DM.
  - Organise a health day where the community is screened for DM by nurses, and those with risk factors are tested with a glucometer.
  - Have a mobile health clinic with DM screening and testing at any large community gatherings.
- High prevalence of obesity (2)
  - Form support groups for weight loss or exercise clubs in collaboration with NGOs (Weight Watchers) and departments of sport and recreation.
  - Offer weighing/BMI/waist circumference screening and interpretation during door-to-door campaigns, CHW home visits, health days, or a mobile clinic at community activities.
  - Organise a fun run, walk, or marathon to raise awareness about DM.
  - A cooking club promotes healthy recipes, so develop a local healthy recipe book.

**Further reading**

- Marcus T, Hugo J. Chapter 7: Community-orientated primary care: Where there is a doctor. In Mash B, editor. Handbook of Family Medicine (4th ed). Cape Town: Oxford University Press; 2017, 334-359.

## Critical appraisal of quantitative research

Read the accompanying article carefully and answer the following questions. As far as possible, use your own words. Do not copy out chunks from the article. Be guided by the allocation of marks concerning the length of your responses.

- Essop MR, Seedat F, Raal FJ. Dyslipidaemia in patients with chronic kidney disease – A neglected cardiovascular risk factor. S Afr Med J. 2023;113(11):1479–1484. https://doi.org/10.7196/SAMJ.2023.v113i11.1089

**Total: 30 marks**

1. Did the study address a focused issue? (4 marks)
2. Critically appraise the authors’ choice of study design for addressing the research question. (3 marks)
3. Critically appraise the recruitment strategy. (5 marks)
4. Were the exposure and outcomes of interest accurately measured validly and reliably? (4 marks)
5. Critically appraise the statistical test choice for this study. (4 marks)
6. Discuss the dataset’s completeness and the missing data implications on interpreting the study findings. (4 marks)
7. Use a structured approach (e.g. relevance, education, applicability, discrimination, evaluation, reaction [READER]) to discuss the value of these findings to your practice. (6 marks)

**Suggested answers**

### 1. Did the study address a focused issue? (4 marks)

- As the main aim, the authors wished to describe the prevalence, severity and pattern of dyslipidaemia among chronic kidney disease (CKD) patients in a quaternary South African hospital.
- This main aim describes the condition of interest (epidemiological considerations of dyslipidaemia) in a particular subset of patients (those with a chronic renal condition) in a specific context (a quaternary hospital in South Africa).
- Secondary objectives described possible outcomes concerning the condition of interest, such as determining the proportion of CKD patients on lipid-lowering therapy (LLT), whether CKD patients on LLT were at appropriate targets, and evaluating the type and dose of LLT used to treat dyslipidaemia.
- In summary, the answer to the question is affirmative, as the study did aim to address a focused issue in terms of the population studied and the risk factor studied (the condition of interest, dyslipidaemia). The secondary objectives described above provide more details on the outcomes of interest, especially on the efficient use of LLT in this group of patients.

### 2. Critically appraise the authors’ choice of study design for addressing the research question. (3 marks)

- The authors wished to determine the prevalence, severity and pattern of dyslipidaemia in this group of patients, which means that the choice of study design was appropriate.
- Unlike cohort studies, cross-sectional studies are used to determine prevalence. Cohort studies are used to study causes and incidence. Cross-sectional studies are not able to establish a cause-and-effect relationship (causality).
- In this study, the retrospective cross-sectional study design also represented a time- and cost-efficient approach to address the study objectives, and data are readily available from existing patient records attending the quaternary hospital’s renal clinic.

### 3. Critically appraise the recruitment strategy. (5 marks)

- The researchers chose to review the files of patients with CKD attending the quaternary hospital’s renal clinic between 01 July 2019 and 31 July 2020. Members of the research team were based at this facility.
- No clarification was provided to justify the sample size of 250 patients. Usually, a sample size calculation is needed to ensure that the study is sufficiently powered to detect statistically significant differences or effect sizes.
- The criteria for inclusion were well-defined and consisted of documented CKD, adults older than 18 years, and the presence of a lipogram performed in the past year. Interestingly, patient records were selected randomly based on the fulfilment of these inclusion criteria and the completeness of clinic records.
- This recruitment process may have potential selection biases, as the need for complete records was not specified as an inclusion criterion. One understands that the nature of a retrospective review necessitates complete records. However, the existing data may still be biased even if the records were complete. The visit-related data collection, data entry and quality assurance were not planned and were part of routine healthcare practices.
- Furthermore, the data period of 13 months was not justified. This data period also overlapped with the first wave of the coronavirus disease 2019 (COVID-19) pandemic, which may have altered access to routine care and investigations, thereby skewing the dataset to include patients who accessed routine care before the onset of the pandemic and who were able to access care despite the lockdown restrictions.

### 4. Were the exposure and outcomes of interest accurately measured validly and reliably? (4 marks)

- Lipid-lowering therapy would be viewed as the key exposure, and this variable was used to stratify the patient cohort into those on and not on LLT, as demonstrated in Table 1 and Figure 1 in the article.
- The authors reported that ‘the use of immunosuppressive agents, steroids and LLT was noted, in addition to the dose and duration of treatment’. However, as noted in the study limitations section, the documentation of LLT prescription does not equate to actual usage or adherence to LLT. Although the authors used objective measurements, a gap may have resulted in measurement bias.
- The outcomes of interest were treatment targets as specified by the various guidelines, mainly linked to the relationship between the lipogram results and renal function, especially the estimated glomerular filtration rate (eGFR). These laboratory values are usually based on valid and reliable laboratory procedures and protocols and may be considered objective measurements.
- The clinic files were used to obtain socio-demographic information and whether the patient was on renal replacement therapy. Information on comorbidities and complications was also obtained from the patient records. These measurements may seem objective, but extracting the data from the patient records has not been described in terms of whether the whole dataset was captured consistently by a single researcher. The study was a Master’s research project without mention of a research assistant. The authors report using a data collection tool in the methods section, which was transferred into an Excel spreadsheet. If more than one person had captured the data from the 250 files, there may have been some inconsistency in how each person interpreted the clinical records written by other healthcare staff members.

**TABLE 1:** Marking template for objectively structured clinical examination consultation station scenario.

Competencies		Candidate’s rating		
		Not competent	Competent	Good
1.	Establishes and maintains a clinician-patient relationship	-	-	-
2.	Gathering information: history, examination, investigations	-	-	-
3.	Clinical reasoning	-	-	-
4.	Explaining and planning	-	-	-
5.	Management	-	-	-

### 5. Critically appraise the statistical test choice for this study. (4 marks)

- In the methods section, the authors describe the involvement of a statistician and their planned use of descriptive statistics to describe the demographic and clinical profiles of the included patients, with frequencies and proportions for categorical data as well as medians and interquartile ranges for continuous variables.
- Table 1 reports *p*-values when comparing the patient cohort stratified by taking LLT but does not provide the tests used to calculate these *p*-values. The data of the continuous variables (age, weight, and year of first visit to the renal clinic) were not normally distributed as median and interquartile ranges were provided.
- Table 3 reports the use of the chi-square (χ^2^) test to calculate the *p*-values when displaying the prevalence of severe dyslipidaemia stratified by different patient characteristics, such as gender, age older than 50 years, ethnicity, risk profiles, CKD stage, HIV status and presence of LLT.
- The inclusion of an inferential test, such as the χ^2^ test, should have been stated in the methods section as part of the data analysis strategy. This represents sound reporting practice. Descriptive statistics aim to describe and summarise data, whereas inferential statistics make inferences and draw conclusions about a population based on sample data. A χ^2^ test determines whether there is an association between categorical variables (i.e. whether the variables are independent or related).

### 6. Discuss the dataset’s completeness and the missing data implications on interpreting the study findings. (4 marks)

- Despite the specification of using complete patient records as an inclusion criterion and ensuring the use of objective measurements, the study limitations reported the issue of missing data points.
- This is a pitfall of using routinely collected data for the study compared to data collected prospectively with a study-specific data-capturing instrument. In this study, the data were collected retrospectively from clinical records.
- The authors have no control over the variables and information bias. The authors might have underestimated or overestimated the prevalence, severity, and pattern of dyslipidaemia among CKD patients as they focused only on the records of patients with complete datasets who were able to access this quaternary renal clinic during the first wave of the COVID-19 pandemic as services may have been affected from March to July 2020 which represent 5 months of the 13-month study window.
- The study needed more power for subgroup analysis, especially as no sample size calculation was reported. The authors also reported a need for more data on baseline pre-treatment lipid levels, as they designed the list of measurements only to include single lipid test results. These factors have impacted the authors’ ability to address the secondary study objectives.

### 7. Use a structured approach (e.g. relevance, education, applicability, discrimination, evaluation, reaction [READER]) to discuss the value of these findings to your practice. (6 marks)

The READER format may be used to answer this question:

- What is its relevance to family medicine and primary care?
- Education – does it challenge existing knowledge or thinking?
- Applicability – are the results applicable to my practice?
- Discrimination – is the study scientifically valid enough?
- Evaluation – given those mentioned above, how would I score or evaluate the usefulness of this study for my practice?
- Reaction – what will I do with the study findings?

*The answer may be subjective but should reflect on the possible changes in the students’ practice within the South African public healthcare system. It is acceptable for the student to suggest how their practice might change within other scenarios after graduation (e.g. private general practice). The reflection on whether all essential outcomes were considered depends on the reader’s perspective (is there other information you would have liked to see?)*.

*A model answer could be written from the family physician’s perspective employed in the South African district health system*:

- R: This study is relevant to the African primary care context, as patients with CKD are commonly encountered. Atherosclerotic cardiovascular disease and dyslipidaemia are equally common and represent a significant burden of illness. Furthermore, having an integrated approach to multimorbidity encapsulates the ethos of person-centred, holistic primary care. A better understanding of dyslipidaemia in patients with CKD in primary care clinics will help primary care teams and policymakers plan appropriate interventions.
- E: The authors cited heterogeneous recommendations of current guidelines on LLT for patients with CKD, which complicates the implementation across all levels of care, especially with the controversy on how best to treat dyslipidaemia to therapeutic target levels within a resource-constrained environment. At the primary care level, however, current standard treatment guidelines on CKD do mention dyslipidaemia and the need for treatment in this high-risk group of patients. This study found a high prevalence of dyslipidaemia in this group of patients with CKD accessing care in a quaternary renal clinic. The authors also discovered that a more significant proportion of patients with CKD did not receive the recommended LLT, and only a small minority achieved the recommended treatment targets.
- A: As this study zoomed in on the practice of a quaternary renal clinic, the study findings may not be generalisable to the primary care context. However, one could consider the care continuum of these patients with CKD and how their care should be coordinated between their primary care teams and specialist care providers. The need for early intervention and risk management related to atherosclerotic cardiovascular disease warrants a proactive approach to disease prevention and health promotion. A high index of suspicion and early intervention is required at the primary care level.
- D: On discrimination, there is a fair congruity between the research methodology, data collection methods and data analysis. An element of selection and measurement bias may impact the dataset and study findings. Furthermore, the results were also impacted by the need for a sample size calculation and the resultant impact on the study’s power to perform subgroup analyses.
- E: The study’s findings may be relevant when considering providing comprehensive care for patients with CKD across the care continuum. It may be helpful to remind oneself of the nature of the study design and its limitations and that the study setting is not representative of primary care practice.
- R: The study’s findings may help create awareness of the need to include dyslipidaemia diagnosis and management in the comprehensive care plan for patients with CKD. The study argued for the need to improve the management of dyslipidaemia in patients with CKD in the South African public sector to reduce the burden of morbidity and mortality in the CKD population. Effective interventions, such as comprehensive pharmacological and non-pharmacological preventative measures, should be implemented in primary care to minimise the impact of undiagnosed and sub-optimally managed dyslipidaemia in patients living with CKD.

**Further reading**

- Pather M. Evidence-based family medicine. In: Mash B, editor. Handbook of family medicine. 4th ed. Cape Town: Oxford University Press, 2017; p. 430–453.
- MacAuley D. READER: An acronym to aid critical reading by general practitioners. Br J Gen Pract. 1994;44(379):83–85.
- The Critical Appraisals Skills Programme (CASP). 2023. CASP checklists [homepage on the Internet]. [cited 2023 Dec 12]. Available from: https://casp-uk.net/casp-tools-checklists/
- Goodyear-Smith F, Mash B, editors. How to do primary care research. Boca Raton, FL: CRC Press, Taylor and Francis Group; 2019.

## Objective structured clinical examination (OCSE) station scenario: Adult medicine

**The station’s objective**

This station tests the candidate’s ability to manage an undifferentiated patient with fatigue.

**Type of station**

Integrated consultation.

**Role player**

Simulated patient: adult male or female.

### Instructions to the candidate

- You are the family physician working at a community health centre.
- Your task: Please manage this patient.
- You do not need to examine this patient. All examination findings will be provided on request.

## Instructions for the examiner

- This is an integrated consultation station in which the candidate has 20 min.
- Familiarise yourself with the assessor guidelines, which detail the expected responses from the candidate.
- No marks are allocated. In the mark sheet (Table 1), tick off one of the three responses for each competency listed. Ensure you are clear on the criteria for judging a candidate’s competence in each area.
- Provide the following information to the candidate when requested: examination findings.
- Please switch off your cell phone.
- Please do not prompt the student.
- Please ensure that the station remains tidy and is reset between candidates.

## Guidance for examiners regarding Table 1

- The aim is to establish that the candidate has an effective and safe approach to managing an undifferentiated patient with fatigue.
- A working definition of competent performance: the candidate effectively completes the task within the allotted time in a manner that maintains patient safety, even though the execution may not be efficient and well structured:
  ■ *Not competent:* Patient safety is compromised (including ethical-legally), or the task is not completed.
  ■ *Competent*: The task was completed safely and effectively.
  ■ *Good*: Besides displaying competence, the task is completed efficiently and in an empathic, patient-centred manner (acknowledges patient’s ideas, beliefs, expectations, concerns or fears).
- Establishes and maintains a good clinician-patient relationship:
  ■ The competent candidate is respectful and engages with the patient in a dignified manner. The good candidate is empathic, compassionate and collaborative, facilitating patient participation in key areas of the consultation.
- Gathering information:
  ■ The competent candidate gathers sufficient information to establish a working diagnosis *(explores mental health, lifestyle, or underlying general medical conditions and screens for anaemia and diabetes, screens for tuberculosis and HIV)*.
  ■ The good candidate additionally has a structured and holistic approach (*explores psychosocial issues – concern about underlying medical problems and occupational impact; issues in lifestyle that impact sleep and wake cycle; use of drugs or alcohol; specific symptoms of undiagnosed diabetes, anaemia or hypothyroidism)*.
- Clinical reasoning
  ■ The competent candidate identifies the diagnosis (*fatigue possibly due to the underlying medical problem)* and acknowledges *the ongoing psychosocial issues that may be contributory*.
  ■ The good candidate additionally makes a comprehensive biopsychosocial assessment that may be more biomedically specific (*suspects hypothyroidism, may consider long COVID-19*) and expands on contributory psychosocial issues (*ongoing work-related stress, poor sleeping patterns, sedentary lifestyle*).
- Explaining and planning
  ■ The competent candidate uses clear language to explain to the patient that no clear answer is immediately available but needs further investigations and uses strategies to ensure patient understanding (*questions OR feedback OR reverse summarising*).
  ■ The good candidate additionally ensures that the patient is actively involved in decision-making, paying particular attention to knowledge-sharing and empowerment.
- Management
  ■ The competent candidate proposes appropriate intervention. *(TSH and T3 or T4 if abnormal; haemoglobin and white blood cell count [as a minimum]; sleep hygiene trial)*.
  ■ The good candidate additionally discusses *relevant therapeutic interventions if blood results are abnormal, non-pharmacological interventions (exercise, stress management, psychological counselling), and encourages a family or relationship-oriented approach*.

## Role player instructions

Adult male.

Opening statement: ‘Doctor, I have been very tired and am worried that I have some serious medical problem…’.

Open responses: Freely tell the doctor …:

- You are 34 years old.
- Been feeling abnormally tired for the last month or so.
- Now you are worried that you have a medical problem.
- The fatigue is starting to affect your work, and you are very worried about this, as you are studying part-time while working.

Closed responses: Only tell the doctor if s/he brings this up:

- You have no medical problems that you know of. You have never been admitted to the hospital and have no allergies.
- You had the “flu” about two weeks ago, but the tiredness was there before the “flu”.
- This tiredness affects your physical energy and concentration at work. It feels as if you have never had enough sleep. There is no shortness of breath, and you are able to do physical activity when needed.
- Work/Studies: You have been a data collector for an insurance company for the last 3 years. You are studying for an online, part-time certificate in data management. The studies started 5 months ago and will be finishing this month.
- Daily pattern: Work from 07:30 to 15:30. You study every day from 17:00 to 19:00. Usually, you have assignments that you do over the weekend. Engage with family from 19:00 until bedtime for the kids (20:00), then dinner and watch TV until 23:00. The wake-up time is 04:30.
- Family: You are married with two young children (2 and 4 years old). Your wife also works a demanding job.

Family history: Your parents are both alive (63 and 68 years old – healthy). You are an only child.

*Concerns:* you are very concerned about the impact on your work. You are very ambitious and love your studies. If you do well in this course, your managers will identify you for promotion.

## Examination findings

General: well-nourished adult male, lean build. Neat. No jaundice, pallor, clubbing, cyanosis, oedema or lymphadenopathy.

**Vital signs:**

- Blood pressure: 135/85 mmHg.
- Peripheral pulse is easily palpable, with a regular rate of 84 beats/min.
- Temperature: 36.5 °C.
- Weight of 75 kg, height of 1.72 m.

**Systemic examination findings:**

- Skin – no noticeable rash, slightly dry over extremities
- Respiratory – no abnormalities
- Cardiovascular – no abnormalities
- Neurological – no abnormalities
- Musculoskeletal – no abnormalities

**Side room investigations:**

- Urine dipsticks – no abnormalities
- Haemoglobin – 12.5 gm/dL
- Random blood glucose – 6.2 mmol/L.

**Further reading:**

- Department of Health, South Africa. Standard treatment guidelines hospital level, adult [homepage on the Internet]. 2019 [cited 2023 Dec 12]. Available from: https://knowledgehub.health.gov.za/elibrary/hospital-level-adults-standard-treatment-guidelines-stgs-and-essential-medicines-list-eml
- Department of Health, South Africa. Standard treatment guideline primary level. 2020:9.6.3 [homepage on the Internet]. [cited 2023 Dec 12]. Available from: https://knowledgehub.health.gov.za/elibrary/primary-healthcare-phc-standard-treatment-guidelines-stgs-and-essential-medicines-list-eml

